# Describing Dataset Archetypes

**DOI:** 10.1016/j.patter.2020.100168

**Published:** 2020-12-11

**Authors:** Leigh Dodds

**Affiliations:** 1 Open Data Institute, London N1 9AG, UK

## Abstract

The FAIR principles need to be applied in context. To do that, we need to understand both the needs of data users and the characteristics of the data to be shared. This Opinion introduces ten different dataset archetypes that can be used to inform plans for how data are to be accessed, used, and shared.

## Main Text

Many communities across research, industry, government, and beyond are engaged in debates about how to maximize value from data while minimizing harmful impacts from their collection and use.

At the center of those debates is a range of social, legal, and economic issues. What are the appropriate governance models that should guide how sensitive data are being shared? What legal terms of use can help to drive innovation and reuse of data? What are the ethical and privacy implications of collecting and sharing different types of data? Where are the barriers to increasing access to data? And what infrastructure is needed to ensure sustainable access to data over the long term?

While legislation and policy frameworks govern data use at the national and international levels, there are a variety of other frameworks and sets of principles that are guiding approaches to data ethics, data management, and reuse in different contexts.

The FAIR (findable, accessible, interoperable, and reusable) data principles^1^ are one example. Originally defined by researchers working in the life sciences, the FAIR principles have been rapidly adopted across research communities and are being increasingly referenced in other areas. For example, they recently surfaced in the UK government’s Geospatial Data Strategy.^2^

But the FAIR principles, like any framework, need to be applied in context. To do that successfully requires an appreciation of the data ecosystem^3^—the infrastructure, actors, and data flows—in which data are being used. Focusing on a specific ecosystem can help to ground recommendations about how to manage data using specific standards, policies, and practices, and, most importantly, meet the needs of the people represented in or creating value from data.^4^ Data can be FAIR, but we must also ask, for whom?

In user-centered design, tools like user personas^5^ help us to develop pen portraits of a range of typical data users and other actors to better understand their needs and support the creation of well-designed infrastructure and policies.

But it is also important to recognize the variety of different types of datasets that are being collected and shared within those ecosystems. Large-scale datasets collected from Earth observation satellites, streamed from air quality sensors, or collected through medical research projects all have very different characteristics.

These need different approaches to provide access to them and different levels of investment to make sure access is sustainable over the long term.

To help tease out these requirements, we can apply a similar process to that involved in developing personas, but with the goal of developing “pen portraits” or “archetypes” for different types of datasets.

### The Archetypes

Based on experience of working with a number of different types of datasets from both research and government data portals, I have initially outlined the following ten dataset archetypes. Each archetype includes a brief description that describes some of its features along with some specific examples.

#### The Register

The register is a set of reference data that adds context or structure to other datasets. It might consist of a list of specific things, e.g., locations, cars, and services, or a list of categories or types. In both cases, the entries will have a unique identifier and be accompanied by some basic descriptive metadata.

The register is relatively small, but may grow over time. It is stewarded by an organization tasked with making the data available for others. The steward provides some guarantees around the quality and coverage of the data.

It is commonly used as a means to link, validate, and enrich other datasets and is rarely used in isolation other than in reporting on changes to the size and composition of the register.

Examples include licensed premises, registered doctors or companies, a taxonomy of business types or medical conditions, a statistical geography, and addresses.

#### The Study

The study is a dataset that was collected to support a short-term research project. The researchers collected a variety of new data as part of conducting their study. The dataset is small and focused on a specific-use case, and there are no plans to maintain or update it further as the project is now complete.

The group does not have any ongoing funding to support maintenance of the dataset. The data are provided as is for others to reuse; e.g., to confirm the original analysis of the data or to use it in other studies. The dataset is likely published in a research portal or published alongside the academic papers that reference it.

Examples include water quality samples, field sightings of animals, laboratory experiment results, bibliographic data from a literature review, photos showing evidence of plant diseases, and consumer research survey results.

#### The Sensor Feed

The sensor feed is a stream of sensor readings that are produced by a collection of sensors that have been installed across a city. New readings are added to the stream at regular intervals. The feed has been provided to allow a variety of applications to tap into the raw sensor readings for research and commercial purposes.

The data points in the feed are directly reported from the individual sensors and are not quality controlled. The individual sensors may be updated, recalibrated, or replaced over time. The readings are part of the operational infrastructure of the city, so they are be expected to be available over at least the medium term. This means the dataset is effectively unbounded: new observations will continue to be reported until the infrastructure is decommissioned.

Examples include air quality readings, car park occupancy, footfall measurements, rain gauges, traffic light queuing counts, and real-time bus locations.

#### The Statistical Index

The statistical index is intended to provide insights into the performance of specific social or economic policies by measuring some aspect of a local community or economy; for example, a sales or well-being index. The index draws on a variety of primary datasets; e.g., on commercial activities, which are then processed according to a documented methodology to generate the index.

The Index is essentially a data product that is stewarded by an organization and expected to be available over the long term. The dataset is relatively small and is reported against specific geographic areas (e.g., from the register; see below) to support comparisons.

The Index is updated on a regular basis; e.g., monthly or annually. Use of the data typically involves comparing across time and location at different levels of aggregation.

Examples include street safety surveys, consumer price indices, happiness indices, and various national statistical indices.

#### The Corpus

The corpus is a collection of resources, e.g., images, text, or video, that are accompanied by metadata that describe them. A corpus dataset may contain large numbers of entries, but these will all be of the same type; e.g., paintings, photographs, and articles.

A corpus will have been curated to support a specific purpose. This might be a relatively short-term goal, e.g., developing a training dataset for analysis, or more long term, e.g., an archival or cultural heritage dataset. The corpus is rarely updated and grows very slowly over time, if at all.

Examples include bibliographic datasets, art collections, facial recognition datasets, and video archives.

#### The Database

The database is a copy or extract of the data that underpin a specific application or service. The database contains information about a variety of different types of things; e.g., musicians, their albums, and their songs.

It is a relatively large dataset that can be used to perform a variety of different types of queries and to support a variety of uses. As it is used in a live service, it is regularly updated, undergoes a variety of quality checks, and is growing over time in both volume and scope.

Some aspects of the database may reference one or more registers or could be considered as registers in themselves.

Examples include geographic datasets that include a variety of different types of features (e.g., OpenStreetMap and MasterMap), databases of music (e.g., MusicBrainz) and books (e.g., OpenLibrary), company product and customer databases, and Wikidata.

#### The Personal Records

The personal records are a history of the interactions of a single person with a product or service. Depending on the service, the data provide insight into the individual person’s activities, lifestyle, or health.

The data are a slice of a larger dataset that contains data for a larger number of people. As the information contains personal information, it has to be secure, and the individual has various rights over the collection and use of the data as granted by GDPR (General Data Protection Regulation) or similar local regulations.

The dataset is relatively small and focused on a specific set of interactions, but is growing over time. Analyzing the data might provide useful insight to the individual that may help them change their behavior, increase their health, etc.

Examples include bank transactions, home energy usage, fitness or sleep trackers, order history with an online service, location trackers, and health records.

#### The Social Graph

The social graph is a dataset that describes the relationships between a group of individuals. It is typically built up by a small number of contributions made by individuals that provide information about their relationships and connections to others. They may also provide information about those other people; e.g., names, contact numbers, and service ratings.

When published or exported, it is typically focused on a single individual, but might be available in aggregate.

It is a type of dataset different from personal records, as it describes multiple people rather than a history of information about an individual (although personal records may reference or include data about others). The graph as a whole may be maintained by an organization that is operating a social network (or service that has social features).

Examples include social network data, collaboration graphs, and reviews and trip histories from ridesharing services.

#### The Observatory

The observatory is a very large dataset produced by a coordinated large-scale data collection exercise; for example, by a range of Earth observation satellites.

The data collection is intentionally designed to support a variety of different uses, which inform the scale and type of data being collected. That scale can make the dataset difficult to use because of the need to apply specific tools or expertise. But there is a wide range of ways in which the raw data can be processed to create other types of data products, to drive a variety of analyses, or to power a variety of services.

It is refreshed and re-released as required by the needs and financial constraints of the organizations collaborating on collecting and using the dataset.

Examples include Earth observation data, LiDAR (light detection and ranging) point clouds, and data from astronomical surveys or Large Hadron Collider experiments.

#### The Forecast

The forecast is used to predict the outcome of specific real-world events; e.g., a weather or climate forecast.

It draws on a variety of primary datasets that are then processed and analyzed to produce the output dataset. The process by which the predictions are made are well documented to provide insight into the quality of the output. As the predictions are time based, the dataset has a relatively short “shelf life,” which means that users need to quickly access the most recent data for a specific location or area of interest.

Depending on the scale and granularity, forecast datasets can be very large, making them difficult to distribute in a timely manner.

Examples include weather forecasts and outputs of a variety of predictive models.

### A Brief Comparison

As with any characterization, these descriptions lack detail, and the list is not exhaustive. But hopefully they can begin to highlight how planning for FAIR, sustainable, and trustworthy access to data requires some consideration of the differences between datasets.

For example, the Open Definition,^6^ which provides the source definition of open data, indicates that data should be available “as a whole.” However, it is impossible to provide an unbounded dataset like a sensor feed in bulk. And access to large observatory or forecast datasets in bulk may be impractical or unnecessary for some purposes.

Arguments are often made that datasets should be made available via APIs (application programming interfaces) to support their integration into a variety of services and analyses. But for small unchanging datasets like a study, an API is unwarranted. For machine learning applications that use a corpus, dividing it into training and validation datasets, an API-based access model would restrict use unnecessarily.

When it comes to planning for data infrastructure necessary to manage datasets, the complexity and type of infrastructure required to archive a large volume of Studies is very different from that required to support the management of more dynamic datasets like databases and sensor feeds, or to protect databases, personal records, and social graphs that contain personal information that needs stronger governance and protection.

And, with the value and utility of derived datasets, like statistical indices and forecasts dependent on the primary sources used in their creation, there is clearly an increased need for better reporting of provenance.

### Conclusion

There are several ways in which we might start applying these archetypes.

If you are working on a data management plan, then mapping out the types of dataset to be collected in your project might help to inform your decision making, helping you to identify appropriate tools and platforms to support collection, maintenance, and access of data, and, importantly, to prioritize where and how to invest time and effort in managing data to maximize value while mitigating harms.

Data infrastructure includes not just data assets, but also the technology, policy, and communities of users that are benefiting from that infrastructure.^7^

To help us plan, build, and fund the data infrastructure necessary over the long term, we need to ensure that it is sustainable and trustworthy. Doing that requires a clear view of not only what types of data assets that infrastructure will host, but also the ecosystems it is intended to support.

Regardless of whether you are a researcher planning how to collect or manage data, or are working toward developing and maintaining data infrastructure to support research, these data archetypes should provide a useful starting point to help you reflect on your plans and the needs of the users of your data.
